# Coprecipitation Strategy for Halide-Based Solid-State Electrolytes and Atmospheric-Dependent In Situ Analysis

**DOI:** 10.1021/acsami.4c03694

**Published:** 2024-05-16

**Authors:** Josanelle Angela V. Bilo, Chung-Kai Chang, Yu-Chun Chuang, Mu-Huai Fang

**Affiliations:** † Research Center for Applied Sciences, Academia Sinica, Taipei 11529, Taiwan; ‡ Department of Engineering and System Science, National Tsing Hua University, Hsinchu 30013, Taiwan; § Nano Science and Technology Program, Taiwan International Graduate Program, Academia Sinica and National Tsinghua University, Hsinchu 30013, Taiwan; ∥ Department of Science and Technology, Philippine Textile Research Institute, Taguig City 1631, Philippines; ⊥ National Synchrotron Radiation Research Center, Hsinchu 300, Taiwan

**Keywords:** coprecipitation, halide-based solid-state electrolytes, atmospheric-dependent in situ XRD, temperature-dependent in situ XRD, real-world monitoring

## Abstract

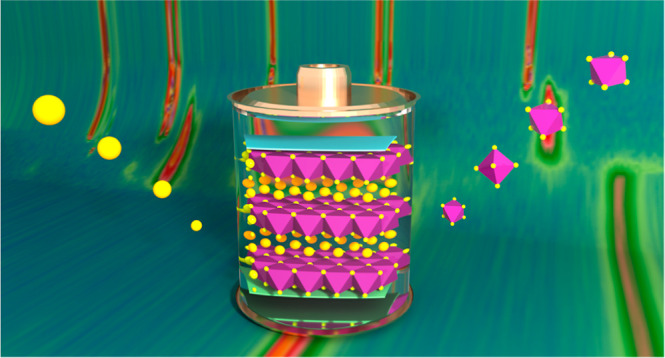

In the continuous pursuit of an energy-efficient alternative to the energy-intensive mechanochemical process, we developed a coprecipitation strategy for synthesizing halide-based solid-state electrolytes that warrant both structural control and commercial scalability. In this study, we propose a new coprecipitation approach to synthesized Li_3_InCl_6_, exhibiting both structural and electrochemical performance stability, with a high ionic conductivity of 1.42 × 10^–3^ S cm^–1^, comparable to that of traditionally prepared counterparts. Through the in situ synchrotron X-ray diffraction technique, we unveil the stability mechanisms and rapid chemical reactions of Li_3_InCl_6_ under dry Ar, dry O_2_, and high-humidity atmosphere, which were not previously reported. Furthermore, the fast reversibility capability of moisture-exposed Li_3_InCl_6_ was tracked under vacuum, revealing the optimal recovery conditions at low temperatures (150–200 °C). This work addresses the critical challenges in structural engineering and sustainable mass production and provides insights into chemical reactions under real-world conditions.

## Introduction

Ternary halides, denoted as Li–M–X (where M represents a metal element, and X is a halogen), have garnered significant attention in halide-based solid-state electrolyte (SSE) research. This heightened interest stems from their advantages: high ionic conductivity (>10^–4^ S cm^–1^), direct compatibility with high voltages, and a wide electrochemical window surpassing 4 V.^1−4^ Among the increasingly reported Li–M–X SSEs, those based on the Li–In–Cl system possess reliability, demonstrating good ionic conductivity at room temperature. However, the practical application of halide-based SSEs in solid-state batteries necessitates a facile and cost-effective synthesis route to achieve high ionic conductivity. Most halide SSEs were synthesized through energy-intensive mechanochemical synthesis with high-temperature annealing processes^5−8^ and solid-state reactions,^3,4,9^ similar to its sulfide and oxide SSE counterparts,^10−12^ posing challenges in obtaining uniform-size powder SSEs and hindering large-scale commercial production. To address these limitations, a more energy-friendly liquid-mediated synthesis has become a viable alternative, aiming for homogeneity and easy scalability.^13−15^ The wet-chemistry route, specifically the water-mediated synthesis, is recognized as a promising strategy due to its eco-friendly nature, cost-effectiveness, and potential for practical applications.^15^ However, this approach has been limited to the synthesis of Li_3_InCl_6_ from an aqueous solution, primarily attributed to reversible interconversion between the hydrated intermediate and dehydrated phase.^16^ Therefore, continually exploring different strategies to establish a general liquid-mediated method and an in-depth understanding of the chemical reactions involved in liquid synthesis are crucial for developing various halide-based SSEs.

**Figure 1:**
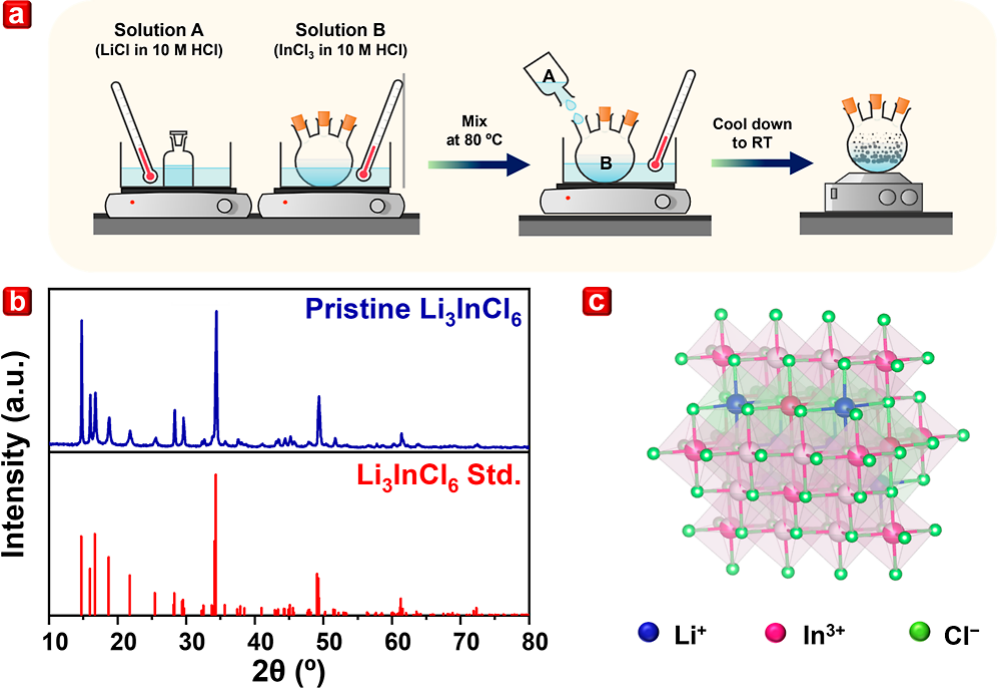
(a) Schematic illustration of the coprecipitation synthesis of Li_3_InCl_6_ SSE, (b) XRD pattern, and (c) crystal structure of pristine Li_3_InCl_6_.

Here, we propose a coprecipitation strategy to synthesize Li_3_InCl_6_. This one-pot approach is achieved by high Cl^–^ concentration through the “common-ion effect” and immediately precipitated when the precursors are mixed. The illustration of the proposed synthesis route is shown in Figure 1a, and the coprecipitation of Li_3_InCl_6_ with a high-concentration HCl aqueous solution is given as follows
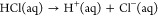

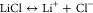

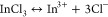

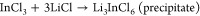
1

The Li_3_InCl_6_ precursor obtained through coprecipitation (1) was washed with hexane to eliminate HCl (aq) traces. After hexane was fully evaporated using a vacuum oven, the Li_3_InCl_6_ precursor attained a powder state. However, the corresponding X-ray diffraction (XRD) patterns did not match the Li_3_InCl_6_ reference, as illustrated in Figure S1. To ensure that we also eliminate any trace amounts of water from the Li_3_InCl_6_ precursor to circumvent the formation of unwanted hydrated phases prior to post-treatment, the vacuum pressure curve of the sintering procedure was monitored every 10 min at 100 °C for 6 h. Figure S2 indicates that after treating the sample at 100 °C for 10 min, a trace amount of water was absorbed by the Li_3_InCl_6_ precursor, as shown by the high initial vacuum pressure of 0.0748 Torr. To address this, the sample was presintered at 100 °C for 6 h under vacuum to remove any absorbed water before undergoing further post-treatment at 200 °C for 4 h. The detailed experimental processes are outlined in the Supporting Information. The obtained Li_3_InCl_6_, after post-treatment, exhibited a pure phase well-indexed to a distorted monoclinic rock-salt structure (Figure 1c) with the *C*2/*m* space group of Li_3_InCl_6_ standard crystallographic information framework (CIF) file in the database (PDF 04-009-9027), as shown in Figure 1b.^5,17^ Moreover, high-resolution synchrotron powder XRD (Figure S3) further elucidated the structural properties of Li_3_InCl_6_.

## Results and Discussion

### Structural Analyses

**Figure 2:**
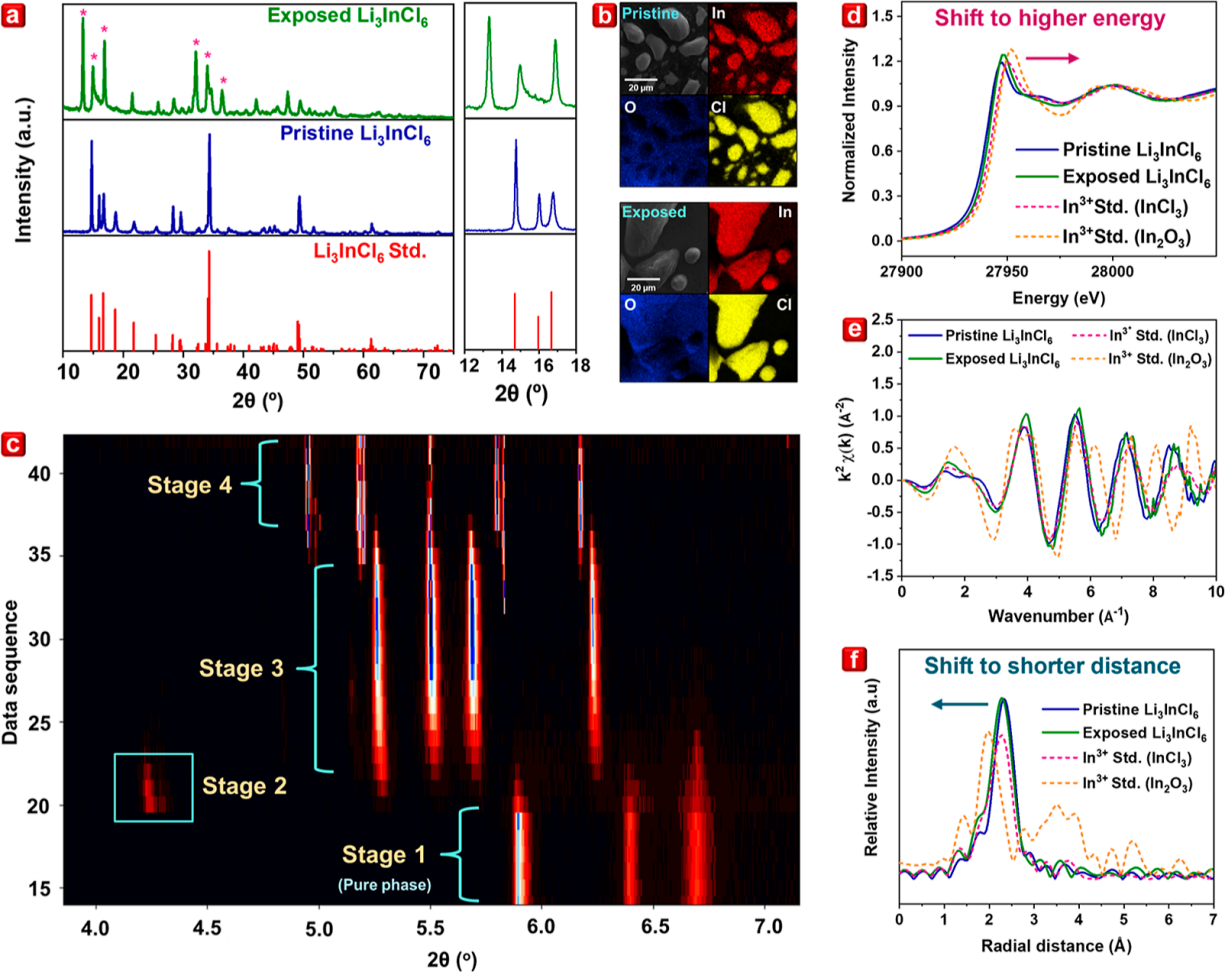
(a) XRD profile and (b) EDS images at 3000× magnification of pristine Li_3_InCl_6_ and Li_3_InC_l6_ exposed to ∼60% relative humidity for 30 s. (c) Contour plot of the moisture-dependent in situ XRD exposed to 100% relative humidity. (d) In *K*-edge XANES spectra, (e) In *K*-edge *k*^2^-weighted Fourier transforms of EXAFS spectra, and (f) In *K*-edge *R*-space EXAFS spectra of pristine Li_3_InCl_6_ and Li_3_InCl_6_ exposed to ∼60% relative humidity for 3 min.

One of the critical factors influencing halide-based SSE’s performance is the presence of moisture in the air, particularly significant for Li_3_InCl_6_ due to its high deliquescence, attributable to its relatively smaller cationic radius (*r*_In_^3+^ = 80 pm).^18^ To ensure consistency, pristine Li_3_InCl_6_ was intentionally exposed to ∼60% relative humidity for 30 s throughout the moisture-effect experiments. As depicted in Figure 2a, the XRD pattern of Li_3_InCl_6_ upon exposure to ∼60% relative humidity for 30 s cannot be indexed with the Li_3_InCl_6_ reference and its possible degradation products such as InOCl, InCl_3_, LiCl, and hydrated Li_3_InCl_6_. To comprehensively investigate the changes in the structural properties of the pristine Li_3_InCl_6_, in situ moisture-dependent synchrotron powder XRD was carried out using a synchrotron-based X-ray with a photon energy of 20 keV. The pristine Li_3_InCl_6_ was purged with Ar gas and exposed to moisture (100% RH). Each data point was recorded every 5 s with a 5 s resting time. This technique enables rapid scanning and penetration of samples, providing comprehensive crystal structural information for the entire material without slowing the chemical reaction by decreasing the percentage of humidity, thus mimicking the real-world monitoring of the chemical reaction of halide-based SSEs with air moisture. Continuous synchrotron XRD spectra were collected over time throughout the moisture exposure process, and the observable changes in the XRD pattern of the pristine Li_3_InCl_6_ became evident, indicating the reaction of the pristine Li_3_InCl_6_ with air moisture. The contour plot in Figure 2c illustrates the four distinct stages of structural evolution observed during this process. Stage 1 corresponds to pristine Li_3_InCl_6_. In stage 2, structural changes are evident, as indicated by the emergence of several peaks at around 4.3°, signifying moisture-induced degradation; these peaks may be a combination of different phases. One possible impurity can be attributed to InOCl; however, the entire crystal structure cannot be fully resolved due to the lack of a single crystal in these states. Moreover, this impurity is transient, lasting only a few seconds (stage 2), representing an intermediate state before progressing to stage 3. To the best of our knowledge, the impurities in stage 2 have not been detected previously, thus highlighting the power of the quick-scan synchrotron XRD technique employed in our study. This short-lived intermediate necessitates further in-depth investigation as it might initiate the instability in halide-based SSEs when exposed to air moisture.

Moreover, the XRD profile of stage 3 lasted for several minutes before it transitioned to stage 4, which was a completely different phase. Neither stage 3 nor stage 4 can be indexed with any known degradation products of Li_3_InCl_6_, suggesting a complete transformation in the crystal phase of Li_3_InCl_6_ at these stages.

### Local Structural Analyses

Furthermore, the X-ray absorption spectroscopy (XAS) technique is employed to determine the locally coordinated environment of the selected ion throughout the bulk material.^19,20^ The XAS spectra, including the X-ray absorption near edge structure (XANES) and extended X-ray absorption fine structure (EXAFS) spectra, of pristine Li_3_InCl_6_ and Li_3_InCl_6_ exposed to ∼60% relative humidity for 3 min are shown in Figure 2d–f. Figure 2d presents the In *K*-edge XANES spectra of the Li_3_InCl_6_ sample. Comparative analysis with pristine Li_3_InCl_6_ and exposed Li_3_InCl_6_ In *K*-edge XANES reveals a slight shift in absorption edge toward higher energy in the exposed Li_3_InCl_6_. This observation suggests an alteration in the local chemical environment surrounding In ions in Li_3_InCl_6_ when they are exposed to air humidity. This inference is further substantiated by In *K*-edge *k*^2^-weighted Fourier transforms of EXAFS, as indicated in Figure 2e. The oscillation patterns observed for exposed Li_3_InCl_6_ deviate slightly from the pristine Li_3_InCl_6_ oscillation pattern, indicating a distorted local structure around In due to the changes in the local environment when Li_3_InCl_6_ was exposed to air moisture. Moreover, the In *K*-edge *R*-space EXAFS spectra of exposed Li_3_InCl_6_ reveal that the distance between the In ion and its first coordinated shell is longer than that of the In^3+^ standards (InCl_3_ and In_2_O_3_). This discrepancy is accompanied by a slight shift toward a lower radial distance, as shown in Figure 2f, which aligns with the shorter bond length of In–O (2.19 Å)^21,22^ compared to that of In–Cl (2.52 Å).^23^ This information indicated that upon exposure to moisture in the air, certain In–Cl bonds might break, which may result from the exposure of In ions to the oxygen ions in the local chemical environment, thereby causing the shift in the lower radial distance. This is further substantiated by the energy-dispersive X-ray spectroscopy (EDS) profile of exposed Li_3_InCl_6_ shown in Figure 2b, revealing the oxygen element within the powder sample.

### In Situ Moisture- and Atmospheric-Dependent Analyses

**Figure 3:**
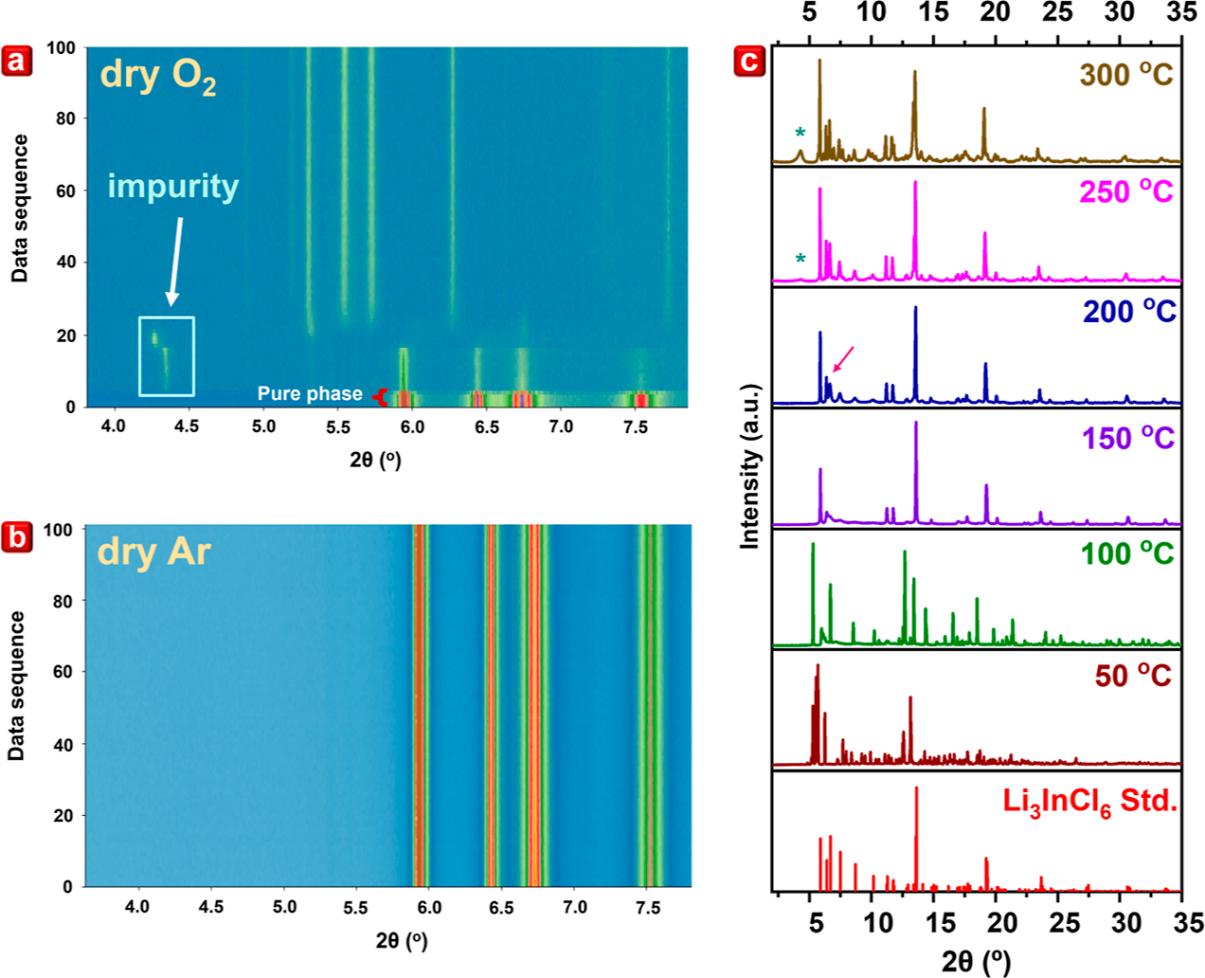
Contour plot of the in situ synchrotron XRD study of pristine Li_3_InCl_6_ when exposed to (a) dry O_2_ and (b) dry Ar and (c) in situ temperature-dependent synchrotron XRD spectra evolution of exposed Li_3_InCl_6_ at different temperatures. The temperature is increased from 50 to 300 °C at a ramped rate of 0.2 °C/s with an exposure time of 3 s.

Given that the substantial influence of the processability and stability of Li_3_InCl_6_ is also crucial as they significantly impact manufacturing processes and associated costs, it is also imperative to assess the stability of the Li_3_InCl_6_ SSEs synthesized via coprecipitation under various gas environments such as dry O_2_ and dry Ar. This assessment is necessary as battery assembly, storage, and electrochemical performance characterization are conducted in an Ar-filled glovebox. Moreover, understanding the resilience of halide SSEs to oxygen is pivotal in reducing the need for rigorous atmospheric controls during large-scale fabrication and battery design. Here, atmosphere-dependent synchrotron XRD is utilized to investigate the stability of Li_3_InCl_6_ by subjecting the sample to a continuous flow of dry O_2_ (99.999%) at room temperature at a flow rate of 20 sccm. High-purity gases were used and passed through molecular sieves throughout the experiment to eliminate any potential moisture influence. The experiment commenced with pristine Li_3_InCl_6_, as shown in the contour plot of dry O_2_ and dry Ar in Figure 3a,b, respectively. The XRD pattern of the sample was acquired every 7 s with a 1 s rest between scans to get a total of 100 scans. Notably, during the exposure to dry 99.999% O_2_ gas, some impurities in the lower angles began to appear after a few scans, as evidenced in the contour plot in Figure 3a, similar to the transient impurity observed in stage 2 of Figure 2c. This observation suggests the inherent instability of pristine Li_3_InCl_6_ in the presence of dry O_2_. To confirm that this effect is not due to the failure of molecular sieves to trap any potential water vapor, the atmosphere-dependent synchrotron XRD of pristine Li_3_InCl_6_ exposed to dry 99.999% Ar gas is characterized, as shown in Figure 3b. However, there were no apparent changes in a total of 100 scans. This result confirmed that molecular sieves effectively filtered out moisture influence during the atmosphere-dependent synchrotron XRD experiments. Moreover, the contour plots during exposure to dry O_2_ and air moisture illustrated in Figures 3a and 2c, respectively, suggest a significant influence of dry O_2_ and moisture on the degradation mechanism of Li_3_InCl_6_. This observation also aligns with the XRD pattern obtained during the moisture experiment by using in-house XRD (Figure 2a). However, the different degradation stages observed in the in situ moisture experiment were not captured using in-house XRD due to scan rate limitations. Thus, the results demonstrated the benefit of using a quick-scan synchrotron XRD to monitor chemical reactions, especially the ones with fast reaction speed.

To assess the recoverability of moisture-exposed Li_3_InCl_6_, in situ temperature-dependent XRD under ambient air was employed. Throughout the experiment, the temperature was ramped from 50 to 300 °C at a ramped rate of 0.2 °C/s with an exposure time of 3 s for each pattern. The pure-phase Li_3_InCl_6_ was recovered when the temperature reached the range of 150–200 °C, with 200 °C exhibiting the highest crystallinity evident from the XRD patterns that presented a sharp peak (marked by red arrow). However, with the temperature increasing beyond 200 °C, an InOCl impurity (represented by the green asterisk) from the lower angle emerged, as shown in Figure 3c. These results reveal the rapid reversible capacity of Li_3_InCl_6_ at relatively low temperatures (150–200 °C), which was not demonstrated before. However, the recovered Li_3_InCl_6_ has lower ionic conductivity (5.83 × 10^–4^ S cm^–1^) than that of pristine Li_3_InCl_6_, as depicted in Figure S5. This reduction may be attributed to the recovery process, where the sample was heated at 200 °C without redissolving. Such heating likely induced surface cracks in the pellet, detrimentally affecting its ionic conductivity. Nonetheless, this approach remains valuable for identifying the optimal synthesis temperature for SSEs.

### Moisture-Dependent Morphology Analyses

**Figure 4:**
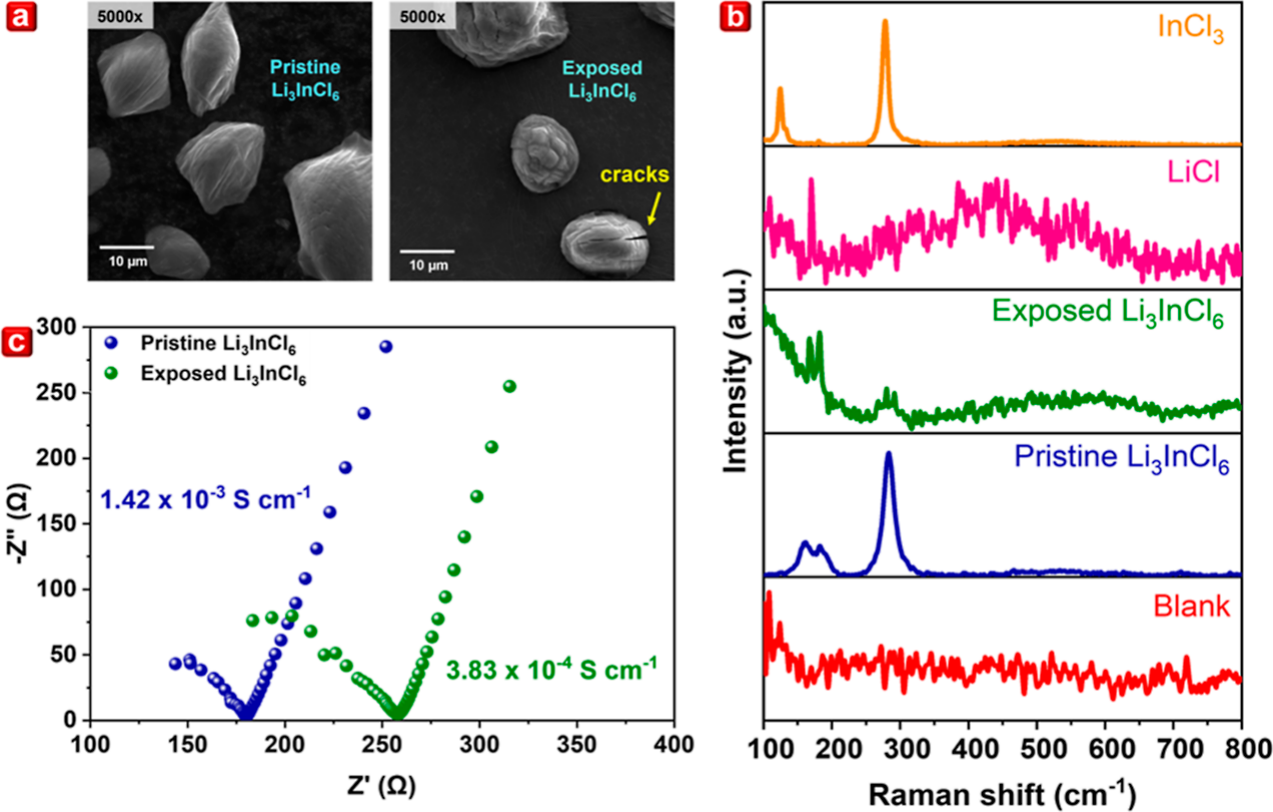
(a) SEM images captured under 5000× magnification, (b) Raman spectra (with LiCl and InCl_3_ and capillary tube), and (c) Nyquist plot of pristine Li_3_InCl_6_ and Li_3_InCl_6_ exposed to ∼60% relative humidity for 30 s.

To substantiate the influence of moisture on the morphology of Li_3_InCl_6_, scanning electron microscopy (SEM) images of pristine and exposed Li_3_InCl_6_ samples were captured under 5000× magnification. As illustrated in Figure 4a, the exposed Li_3_InCl_6_ appears smaller, with particle distribution ranging from 5 to 20 μm. Notably, the surface of exposed Li_3_InCl_6_ reveals observable cracks in contrast to the smoother surface of pristine Li_3_InCl_6_. This discrepancy is attributed to the degradation of the sample during the reaction of Li_3_InCl_6_ with air moisture. Additionally, optical microscopy (OM) was employed to provide insights into the body color, transparency, and impurity distribution within the sample when it is exposed to air moisture, which is not evident in the SEM images (Figure 4a). OM images of pristine and exposed Li_3_InCl_6_ were captured at 1500× magnification. As presented in Figure S4, the pristine Li_3_InCl_6_ powder exhibits a relatively larger particle size and nearly white color with no apparent variation. On the contrary, exposed Li_3_InCl_6_ displays significantly smaller particle sizes and appears transparent, indicating complete moisture absorption by Li_3_InCl_6_ and the formation of liquid beads.

### Raman Spectroscopy and Ionic Conductivity Assessment

Moreover, Raman spectroscopy was carried out to elucidate further the response of pristine Li_3_InCl_6_ to ∼60% relative humidity. The Raman spectra of pristine Li_3_InCl_6_ exhibited a characteristic vibration of InCl_6_^3–^ octahedra. As shown in Figure 4b, the pristine Li_3_InCl_6_ displayed a distinct sharp peak at 281 cm^–1^, corresponding to an In–Cl symmetric stretching vibration at ∼281 cm^–1^. The broad peaks spanning 157–196 cm^–1^ are attributed to In–Cl symmetric bending in-plane and out of the plane, respectively.^24^ Upon exposure to air moisture, the Raman spectrum of Li_3_InCl_6_ resembled that of the precursor (LiCl) spectrum, indicating the partial decomposition of Li_3_InCl_6_ when subjected to moisture. Besides, the Raman spectrum of the capillary tube, used as a sample holder, was measured to ensure that the acquired spectra during the measurement remained unaffected by the sample holder used.

Lastly, Li_3_InCl_6_ synthesized through coprecipitation exhibited a high ionic conductivity of 1.42 × 10^–3^ S cm^–1^, comparable to that of traditionally prepared Li_3_InCl_6_ counterparts.^1,5,8,25^ This similarity is crucial as it underpins stability in both the structural domains and electrochemical performance of the as-synthesized Li_3_InCl_6_. Additionally, we assessed the impact of ∼60% relative humidity on the ionic conductivity of Li_3_InCl_6_, as depicted in Figure 4c. The ionic conductivity of pristine Li_3_InCl_6_ decreases from 1.42 × 10^–3^ to 3.83 × 10^–4^ S cm^–1^ after 30 s of exposure. These results emulate the substantial impact of air moisture on the electrochemical performance of Li_3_InCl_6_ in laboratory settings.

## Conclusions

In summary, we developed a coprecipitation strategy that can be achieved by high Cl^–^ concentration through the “common-ion effect”. This innovative approach can synthesize halide-based SSEs, demonstrating promising potential for scalable commercial production. Notably, within the scope of this research, we successfully applied the coprecipitation method to synthesize Li_3_InCl_6_ and maintain its structural integrity. A quick-scan high-resolution synchrotron XRD was employed to explore the instability mechanisms under various gases (i.e., dry Ar, dry O_2_, and air moisture) and to trace the rapid chemical reaction emulating real-world conditions. Moisture-effect experiments were further assessed by XANES and EXAFS techniques, revealing changes in the local coordination of the In ions of Li_3_InCl_6_ when subjected to air moisture. Moreover, EDS images reinforced the absence and presence of oxygen within Li_3_InCl_6_ upon exposure to air. Collaborative use of OM and SEM provided comprehensive morphological information, including surface structure, size, body color, transparency, and impurity distribution within Li_3_InCl_6_ before and after air moisture exposure. Additionally, the reversible phase transition of the moisture-exposed Li_3_InCl_6_ was elucidated through an in situ temperature-dependent synchrotron XRD under vacuum at temperatures ranging from 50 to 300 °C, revealing fast reversibility capability of Li_3_InCl_6_ at relatively low temperatures (150–200 °C). Lastly, the as-synthesized Li_3_InCl_6_ exhibited a high ionic conductivity of 1.42 × 10^–3^ S cm^–1^, which closely parallels that of traditionally prepared Li_3_InCl_6_, highlighting the effectiveness of the coprecipitation strategy. The noted similarity is imperative as it affirms the structural and electrochemical performance stability of Li_3_InCl_6_. Most importantly, the coprecipitation synthesis strategy employed in this study holds promise for extending its applicability to prepare other halide-based SSEs, with detailed findings to be presented in forthcoming studies. This work provides a new technique for preparing halide-based SSEs through a coprecipitation route, ensuring precise structural control, and offers insights into chemical reactions under real-world conditions while enabling the large-scale sustainable production of high-performance energy storage materials.

## Experimental Section

Anhydrous indium chloride (InCl_3_; 99.99%) was purchased from Nova Materials, and anhydrous lithium chloride (LiCl; 99%) was purchased from Thermo Scientific. Li_3_InCl_6_ was synthesized as follows. In separate containers, saturated solutions of LiCl and InCl_3_ in 10 M HCl were mixed at 80 °C. A white precipitate rapidly formed when the mixed solutions were cooled to room temperature. The precipitate was washed with hexane and centrifuged five times at 2000 rpm for 6 min. Subsequently, the washed precipitate was dried under vacuum conditions at 75 °C until the paste-like precipitate turned to powder Li_3_InCl_6_ precursor. This powder was transferred into the alumina crucibles and placed in the tube furnace. To achieve the desired Li_3_InCl_6_ SSE, the Li_3_InCl_6_ precursor underwent a two-step post-treatment process. Initially, the Li_3_InCl_6_ precursor was preheated at 100 °C for 6 h and then at 200 °C for 4 h under vacuum. The heating and cooling rates were set to 5 °C/min. Upon cooling to room temperature, the as-prepared Li_3_InCl_6_ sample was removed from the tube furnace and transferred immediately to an Ar-filled glovebox to prevent air exposure before characterization.

## Supporting Information Available

Characterization; structural information; pressure change; optical microscopy; and electrochemical impedance spectroscopy (PDF)
