# Supplementary material for: Coprecipitation Strategy for Halide-Based Solid-State Electrolytes and Atmospheric-Dependent In Situ Analysis
Source: ACS Appl Mater Interfaces. 2024 May 16;16(21):27394–9. doi: 10.1021/acsami.4c03694 (PMC11145587; doi:10.1021/acsami.4c03694)
Supplement: Supplementary file 1 — am4c03694_si_001.pdf [file am4c03694_si_001.pdf]

Supporting Information

Co-Precipitation Strategy for Halide Solid-State Electrolyte and Atmospheric-Dependent In-Situ Analysis

Josanelle Angela V. Bilo,[†,§,Φ,Ω] Chung-Kai Chang,[Ψ] Yu-Chun Chuang,[Ψ] and Mu-Huai Fang[†,*]

[†]Research Center for Applied Sciences, Academia Sinica, Taipei 11529, Taiwan

[§]Department of Engineering and System Science, National Tsing Hua University, Hsinchu 30013 Taiwan

[Φ]Nano Science and Technology Program, Taiwan International Graduate Program, Academia Sinica and National Tsing Hua University, Hsinchu 30013, Taiwan

[Ω]Philippine Textile Research Institute, Department of Science and Technology, Taguig City 1631, Philippines

[Ψ]National Synchrotron Radiation Research Center, Hsinchu 300, Taiwan

*Corresponding author Email: fangmuhuai@gate.sinica.edu.tw

CHARACTERIZATION:

X-ray diffraction (XRD) patterns of Li_3InCl_6 samples were performed on a Bruker D8 Advance ECO diffractometer with Cu Kα radiation (λ = 1.54059 Å). To avoid air exposure, a PMMA air-tight holder with a knife was used during the measurement. High-resolution synchrotron XRD patterns were obtained at the National Synchrotron Radiation Research Center (NSRRC, Taiwan) TPS 19A1 beamline with a wavelength of 0.61992 Å and X-ray photon energy of 20 keV. The in-situ temperature-dependent, moisture-dependent, and gas-loading synchrotron XRD experiments were conducted at the NSRRC TPS 19A1 beamline with a wavelength of 0.61992 Å. The temperature-dependent experiment was tuned from 30 to 300 ^{o}C, with a 7-second integration time for each data point. Pristine Li_3InCl_6 was purged with Ar gas and exposed to moisture (100% RH) for the in-situ moisture-dependent experiment. The interval between each data point is 5 seconds with a 5-second resting time. In the gas loading experiment, dry Ar (99.999%) and dry O_2 (99.999%) were passed through the molecular sieve to prevent any residue moisture at a flow rate of 20 sccm, and the interval between each data point is 7 seconds with a 1-second resting time. The X-ray absorption spectra of In, including the X-ray absorption near-edge structure (XANES) and extended X-ray absorption fine structure (EXAFS), are conducted at the NSRRC TPS 44A1 beamline. The scanning electron microscope (SEM) images are collected by ThermoFisher Phenom Pharos, equipped with energy dispersive spectroscopy (EDS). The optical microscope (OM) images are characterized by Keyence VHX-7000. Raman spectra were recorded using a confocal micro-Raman system equipped with a microscope and a Horiba Jobin Yvon Lab Ram Aramis spectrometer with a laser providing excitation light at 532 nm with the 1200 l/mm grating. All spectra were corrected for the spectral response of the instruments. The ionic conductivities of pristine and exposed Li_3InCl_6 SSEs were measured by AC impedance spectroscopy, utilizing a cold-pressed 13 mm diameter Li_3InC_6 pellet with 0.1 cm thickness sputtered with Pt diameter on both sides (0.7 cm diameter). The sputtered pellet was then sandwiched between two stainless steels, assembled in a coin cell, and pressed at 3 tons. The EIS measurement frequency ranged from 1 to 7 MHz using Metrohm Autolab Vionic, equipped with Intello 1.5 software. The impedance results were fitted using Nova 2.1.6 software. The ionic conductivity is calculated based on the impedance results. All coin cell preparation processes were conducted inside an Ar-filled glove box.

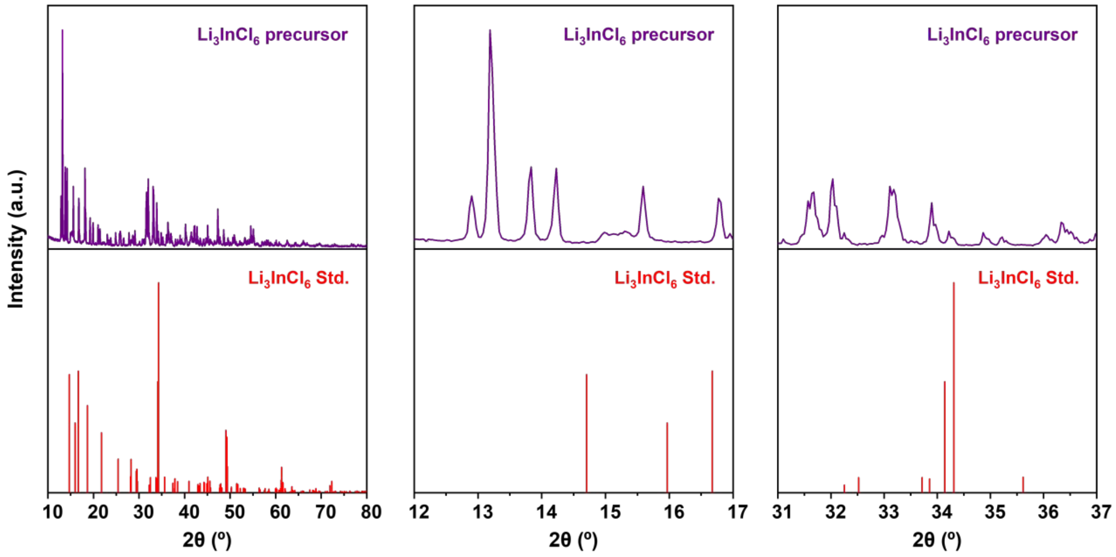

Figure S1. XRD pattern of Li_3InCl_6 precursor before post-treatment.

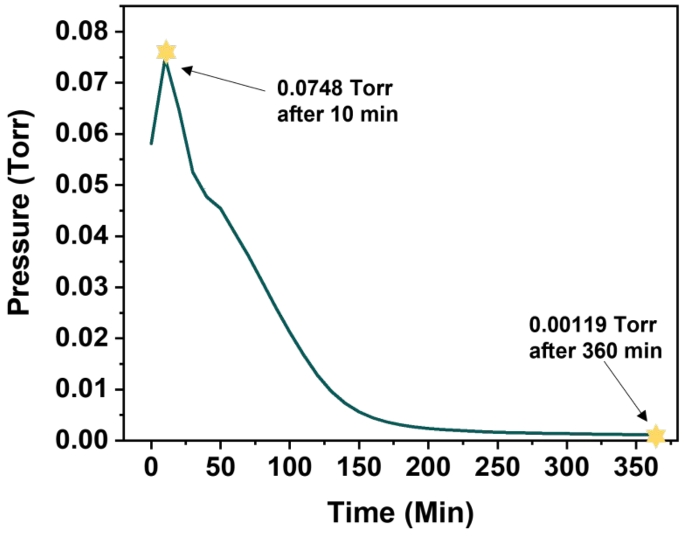

Figure S2. Vacuum pressure curve of Li_3InCl_6 at 100 ^oC

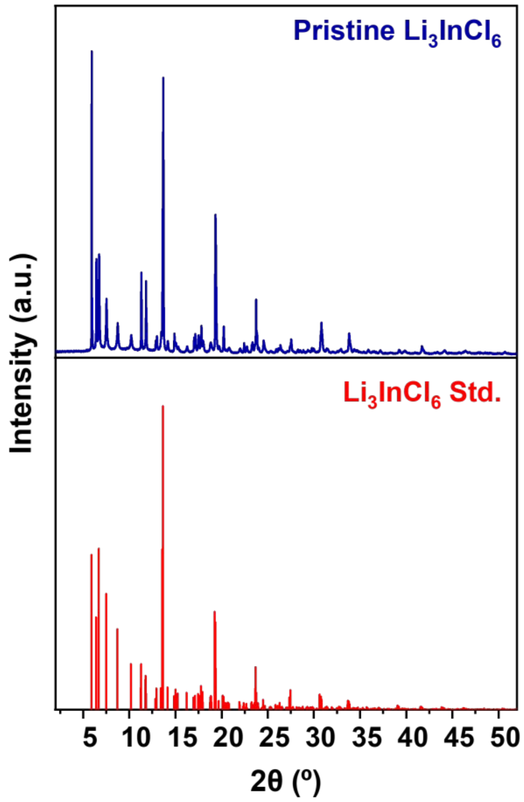

Figure S3. High-Resolution Synchrotron XRD pattern of Li_3InCl_6.

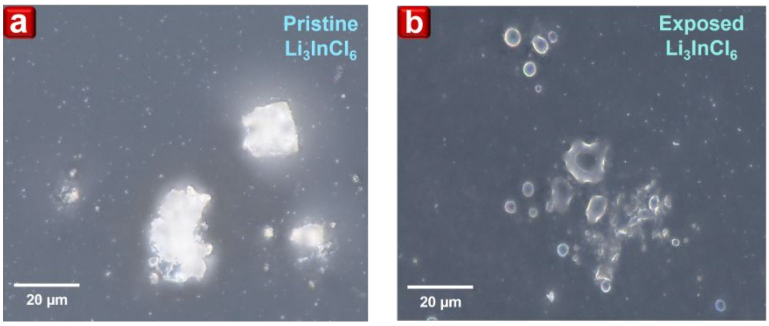

Figure S4. OM images of (a) pristine and (b) exposed Li_3InCl_6 captured under 1500× magnification.

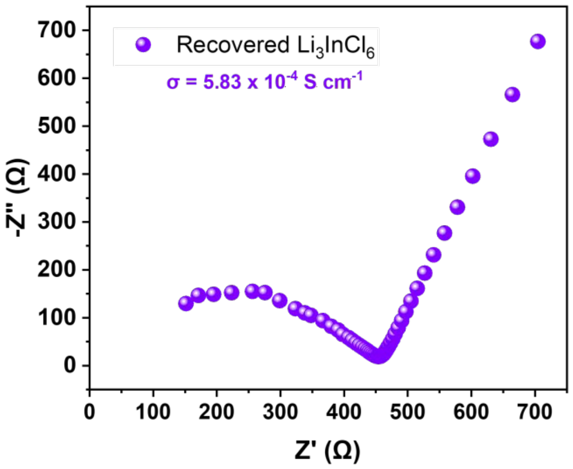

Figure S5. Nyquist plot of recovered Li_3InCl_6.
